# Green synthesis of TiO_2_ nanoparticles using Kinnow peel extracts and their antioxidant properties

**DOI:** 10.1038/s41598-025-22078-z

**Published:** 2025-11-03

**Authors:** Ajay Kumar Tiwari, Abhimanyu Kumar Singh, Saket Jha, Sharad Kumar Tripathi, Ram Raseele Awasthi, Sheo K. Mishra, Rudra Prakash Ojha, Abhishek Kumar Bhardwaj, Anupam Dikshit

**Affiliations:** 1 Department of Physics, G. H. Raisoni International Skill Tech University, Pune, 411006 India; 2 Department of Physics, Shyama Prasad Mukherjee Government Degree College, University of Allahabad, Prayagraj, 211013 India; 3 Department of Surgery, University of Illinois at Chicago (UIC), Chicago, IL 60612 USA; 4 Department of Life Sciences, Arni University, Kangra, 176401 India; 5 Faculty of Engineering and Technology, Khwaja Moinuddin Chishti Language University, Lucknow, 226013 India; 6 Nanoscale Engineering and Sustainable Technology (NEST) Laboratory, Department of Physics, Indira Gandhi National Tribal University, Amarkantak, 484886 India; 7 Department of Biochemistry, University of Lucknow, Lucknow, 226007 India; 8 Department of Environmental Sciences, Amity School of Life Sciences, Amity University Madhya Pradesh, Gwalior, 474005 India; 9 Biological Product Laboratory, Department of Botany, University of Allahabad, Prayagraj, 211002 India

**Keywords:** Kinnow peel extract, Green synthesis, TiO_2_ NPs, DPPH, Antioxidant, Materials science, Nanoscience and technology

## Abstract

The green synthesis of nanomaterials has drawn researchers from all over the world over the past few decades in a huge surge of interest. The aim of this research was to use Kinnow peel extract to synthesize titanium dioxide (TiO_2_) nanoparticles (NPs) in an environmentally friendly and efficient manner. This method seeks to improve antioxidant qualities while using fewer hazardous chemicals in the production of NPs. Using ultraviolet visible (UV–Vis) spectroscopy, the formation of crystalline TiO_2_ NPs was first verified by a distinctive absorbance peak at 235 nm. Further characterization was performed using X–ray diffraction (XRD), field emission–scanning electron microscopy (FE-SEM), energy–dispersive X–ray spectroscopy (EDX), transmission electron microscopy (TEM), and Fourier transform infrared (FT–IR) spectroscopy. The antioxidant potentials of the green-synthesized TiO_2_ NPs were evaluated using DPPH and FRAP assays. The results demonstrated potent free radical scavenging activity, comparable to ascorbic acid, a well-known standard antioxidant. These findings suggest that TiO_2_ NPs possess effective antioxidant properties and highlighting Kinnow peel extract as an eco-friendly sustainable and alternative to conventional synthesis routes. Moreover, the study indicate that the green-synthesized TiO_2_ NPs could serve a promising candidate to replace the conventional antioxidant drugs, such as ascorbic acid.

## Introduction

 Nanoparticles (NPs) are defined as materials with at least one dimension in the nanoscale range of 1–100 nm. Their extremely high surface–to–volume ratio imparts distinctive physical, chemical, and biological characteristics, which are often more effective than those of their bulk materials^1^. Because of these unique features, NPs are considered highly versatile and have been investigated for a variety of applications. Significant research has been carried out on their use in biotechnology, solar cell technology, microbiology, and pharmaceutical development^2–4^. Their distinct physicochemical properties allow for precise molecular-level interactions, while their electrical properties enhance conductivity and sensing capabilities. Moreover, their mechanical strengthening supports the development of a light weight yet robust material^5–7^. The integration of NPs into scientific and medical fields continues to drive innovation, underscoring their indispensable role in modern research and industry. However, chemically synthesized NPs are often deemed unsuitable for pharmaceutical and nutraceutical applications due to their high toxicity, raising serious concerns about human health. This has prompted researchers to explore alternative synthesis methods that are less toxic and more environmentally sustainable. Among these, biological synthesis has emerged as a promising approach for producing NPs with controlled size and morphology. It is regarded as simple, eco-friendly, and cost-effective^8,9^. Plants extract, possess a rich source of biomolecules, such as proteins, polysaccharides, amino acids, organic acids, vitamins, and numerous phytochemicals, including polyphenols, flavonoids, terpenoids, alkaloids, tannins, and alcohol-based compounds. These naturally occurring constituents act as an effective agent, making plant-mediated synthesis an effective route for the green synthesis of NPs^10–12^. Moreover, the preparation of plant extracts is simple and easily scalable, making them suitable for industrial-scale nanoparticle production. Research has demonstrated that plant extracts are often more effective than microbial methods in reducing metal ions, and the resulting NPs have greater stability^13^. Additionally, edible fruits are advantageous because they are safe, non-toxic, and naturally abundant in biomolecules that can act as reducing and stabilizing agents while generating minimal harmful byproducts. Citrus fruits, such as lemon, oranges, limes, tangerines, and grapefruits, contain high levels of bioactive compounds, including citric acid, ascorbic acid, and polyphenols. Their peels are also a valuable source of limonoids, carotenoids, flavonoid glycosides, coumarins, glycosides, glycosides, and essential oils^14^. These compounds collectively exhibit better biocompatibility and contribute to the effective green synthesis of NPs. Recently, researchers have taken an interest in the synthesis of metal and metal oxide NPs through greener routes, owing to their nanoscale dimensions, enhanced durability, and improved stability^15^. Among these, TiO_2_ NPs have gained considerable attention due to their wide range of applications in medicine, environmental remediation, and electronics^16^. TiO_2_ NPs are well–known semiconductors with a band-gap ranging from 3.0 eV to 3.8 eV, which corresponds to their anatase and rutile phases^17^. In contrast, the brookite phase is rare and more difficult to obtain^18^. While the anatase and rutile possess a tetragonal crystal structure, brookite exhibits an orthorhombic arrangement^19^. Due to their versatility, TiO_2_ NPs are widely used in cosmetics as sunscreens for UV protection, photocatalysis, environmental purification, and energy production^20^. Green synthesis of TiO_2_ NPs using plant source extracts, such as citrus fruit peels, offers an environmentally friendly alternative to conventional methods. In this green approach, natural compounds serve as reducing and stabilizing agents, which do not limit only environmental impact but also improve the functional properties of NPs^21^.

Several synthesis techniques, such as sol-gel^22^, electrochemical anodic oxidation^23^, flame synthesis^24^, photoreduction^25^, and thermal hydrolysis^26^, are utilized to synthesize the TiO_2_ NPs. Among them, thermal hydrolysis is considered one of the environmentally benign. Nevertheless, green synthesis remains the most suitable and biocompatible approach compared to the physical and chemical methods^27^. Researchers have successfully employed plant sources, including leaves^28^, roots^29^, and fruits^30^, for the synthesis of TiO_2_ NPs for dye degradation and other applications. Singh et al. (2022) synthesized TiO_2_ NPs using lemon juice extract, and demonstrated enhanced performance in dye-sensitized solar cells without any harmful impact on the environment^31^. Narh et al. (2024) also reported Ag-TiO_2_ NPs by *citrus* sinensis peel extract and observed an increased photodegradation rate of 1.5wt.% for Rhodamine B. Additionally, the study demonstrated that the synthesized material exhibited bactericidal properties against *Staphylococcus aureus and Escherichia coli.*^32^. Trans et al. (2025) prepared TiO_2_ NPs using *camellia sinensis* extract and demonstrated their efficiency in deactivating *S. aureus* and *E. coli.,* as well as in photocatalytic degradation^33^. Similarly, Ouerghi and her colleagues (2022) synthesized TiO_2_ NPs using lemon juice extract and compared the properties of green-synthesized TiO_2_ NPs with those produced chemically. This study also reported that the antibacterial activity of synthesized TiO_2_ NPs was comparable to that of the standard drug Ciprofloxacin^34^. Rahman and his colleagues (2025) synthesized TiO_2_ NPs using lemon juice and observed strong bactericidal potential against *anaerobic* multidrug-resistant (MDR) bacteria, suggesting their potential use as a sanitizer^35^.

Researchers and medical practitioners in India and worldwide have increasingly focused on utilizing fruit waste as a valuable substrate in various industries, including food processing, cosmetics, and pharmaceuticals. This trend is driven by the desire to reduce waste and harness the bioactive potential of fruit byproducts^36–38^. Kinnow (*Citrus reticulata*) is a rich source of bioactive compounds with strong antioxidant properties, which contribute to a healthy lifestyle^39,40^. These peels are not only a source of antioxidants but also contain essential oils, flavonoids, and other phytonutrients that can be effectively utilized in diverse applications. The holistic utilization of Kinnow, including its peels, exemplifies a sustainable approach that maximizes agricultural value while minimizing waste, aligning with the global trend toward eco-friendly industrial practices.

The current study focuses on the cost-effective and eco-friendly green synthesis of TiO_2_ NPs using Kinnow peel extract. The synthesized material was systematically characterized using advanced spectroscopic and microscopic techniques to investigate its properties. The TiO_2_ NPs established with phytochemicals having potential ROS capability are reported. Additionally, the plausible mechanism of TiO_2_ NPs formation using naringin present in the kinnow peel extract is proposed. Our study can be explored further for biomedical applications aimed at minimizing stress-related consequences and supporting human health.

## Materials and methods

### Materials and reagents

Kinnow (*Citrus reticulata*) peels (approximately 500 g) were collected from the local market at Naini, Prayagraj, India. Whatman filter paper (Grade 42) was purchased from Sigma Aldrich. Titanium (IV) oxide (CAS #13463–67–7; molecular weight: 79.90) was purchased from Merck Life Science Pvt. Ltd. Ascorbic acid (CAS number: 50–81–7; molecular weight: 176.12), Ethanol (CAS number: 64-17-5; molecular weight: 46.06), Methanol (CAS number: 67–56–1; molecular weight: 32.04), 2, 2–diphenyl–1–picrylhydrazyl (DPPH: C18H12N5O6; CAS number: 1898–66–4; molecular weight: 394.32) were purchased from Himedia Pvt. Ltd. All chemicals and reagents used in this experiment were of analytical or molecular grade. The synthesis of TiO_2_ NPs and the antioxidant activity assay were carried out at the Biological Product Laboratory, Department of Botany, University of Allahabad, India.

### Preparation of Kinnow peel extract

Kinnow peels were thoroughly washed with tap water and air-dried for 15 days. The dried peels were ground into a fine powder, and 25 g of this powder was boiled with 100 ml of double-distilled water for 2 h until the precipitate turned yellow. A 1:10 aqueous Kinnow peel extract was prepared using a refluxing method. After cooling at room temperature for 4–5 h, the extract was filtered through Whatman filter paper (Grade 42), and the filtrate was collected and stored for further use. This method was adapted from Kureshi et al. (2021)^41^ with some minor modifications.

### Green synthesis of TiO_2_ NPs

Kinnow peel extract was employed for the green synthesis of TiO_2_ NPs. The synthesis procedure was adopted from Tiwari et al. (2022)^42^ with some minor modifications. A total of 50 ml of titanium (IV) oxide solution (0.5 M) was added dropwise to 5 g of aqueous Kinnow peel extract under continuous stirring at 1200 rpm using a magnetic stirrer, maintained at 50 °C for 2 h. The resulting precipitate was centrifuged at 6000 rpm for 30 min and washed 2–3 times with ethanol and double-distilled water to remove impurities. The purified product was then dried in a hot air oven at 200 °C for 48 h.

### Mechanism of green synthesis of the TiO_2_ NPs with Kinnow peel extract

In the green synthesis of TiO_2_ NPs, Kinnow peel extract acts as a natural capping and stabilizing agent, ensuring their stability and dispersion. Although the precise mechanism of TiO_2_ NPs formation has not been fully elucidated, researchers have proposed a plausible pathway. In this mechanism, bioactive constituents of Kinnow peels, particularly ‘naringin’, may play a key role in the reduction of titanium ions and stabilization of the nanoparticles. Initially, TiO_2_ is treated with aqueous NaOH under hydrothermal conditions, and it undergoes a dissolution–precipitation reaction, leading to the formation of sodium titanate phases such as Na_2_O·nTiO_2_ (where *n* = 1–3 depending on reaction conditions). In this process, hydroxide ions attack the Ti–O–Ti lattice of TiO_2_, breaking the bonds and forming soluble titanate species. These species then interact with sodium ions to form layered or tunnel-structured sodium titanates. This intermediate phase serves as a precursor for TiO_2_ NPs, which are typically obtained upon subsequent acid washing and thermal treatment that remove sodium ions and reform the TiO_2_ crystal structure in the anatase or rutile phase. The generalized reaction can be expressed as:

$$\:\text{T}\text{i}\text{O}_2\hspace{0.17em}+\hspace{0.17em}2\text{N}\text{a}\text{O}\text{H}\:\to\:\:\text{N}\text{a}_2\text{O}.\text{n}\text{T}\text{O}_2\hspace{0.17em}+2\text{H}_2\text{O}$$

$$\:\text{N}\text{a}_2\text{O}.\text{n}\text{T}\text{i}\text{O}_2+\text{H}_2\text{O}+\text{H}^+\to\:\:\text{T}\text{i}\text{O}\left(\text{O}\text{H}\right)_2+\text{N}\text{a}^+$$

**Fig. 1:**
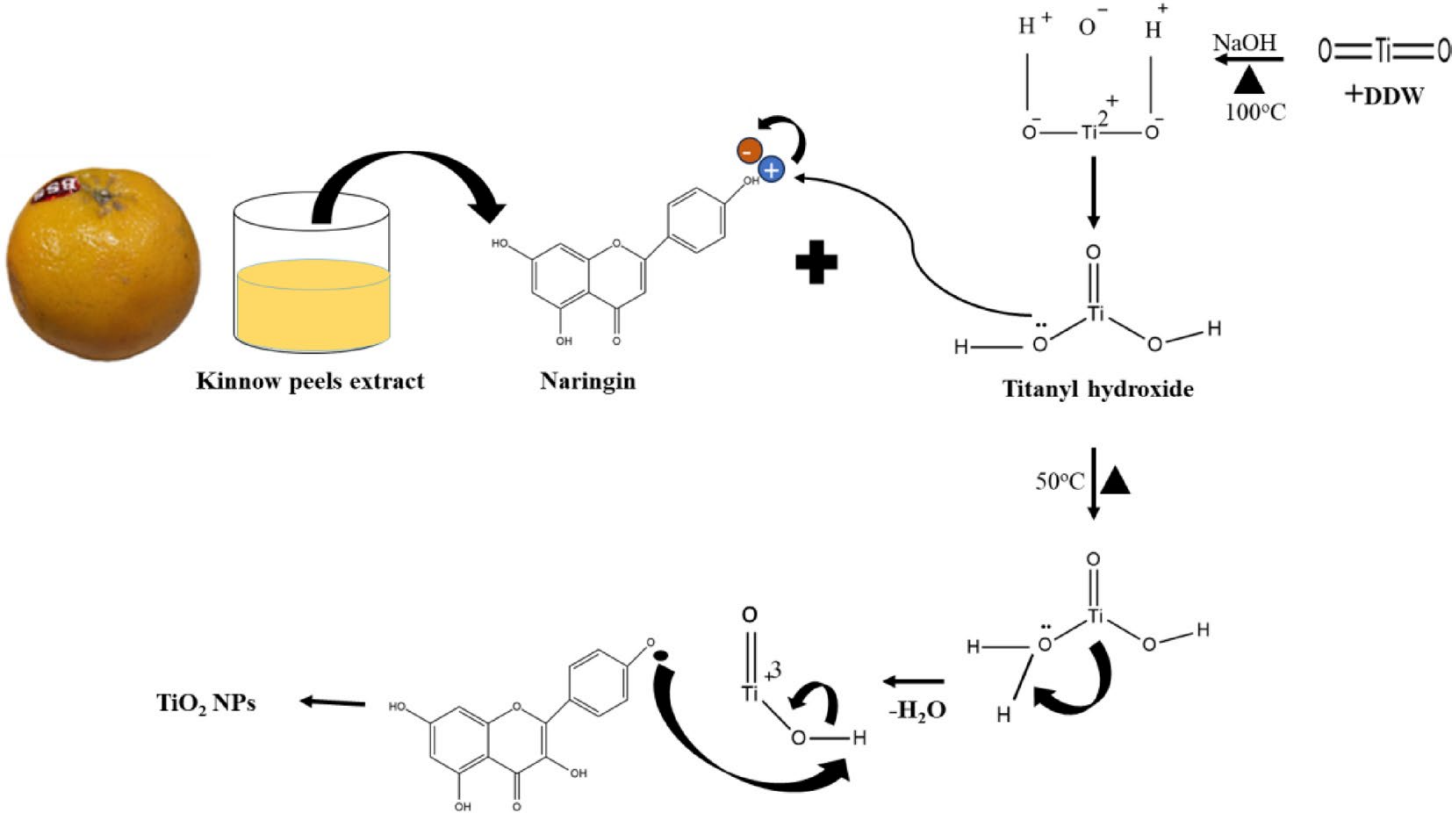
Mechanism of the green synthesis of TiO_2_ NPs.

In other steps, naringin of Kinnow peel extract contains multiple hydroxyl and carbonyl functional groups capable of coordinating with titanium species. Upon mixing with titanyl hydroxide (TiO(OH)_2_) or titanium precursors in an alkaline medium, naringin acts as a complexing agent, forming a Ti–Ti-naringin complex through its phenolic –OH groups. The presence of an electrophilic interaction, titanium is stabilized via ligand-to-metal charge transfer, facilitating the nucleation and growth of TiO_2_ nanostructures^43^, which can be found in a plausible mechanism shown in Fig. 1.

### Characterization of TiO_2_ NPs

The structural properties of green-synthesized TiO_2_ NPs were characterized by X–ray powder diffraction (XRD) with an Ultima IV diffractometer (Rigaku, Japan) over a 2θ range of 20°−70°. The X–ray source was Cu–Kα radiation (λ = 1.54059Å) operated at 40 kV and 30 mA. Surface morphology, particle shape, and sizes were determined from the field emission scanning electron microscopy (FE–SEM) (JEOL JSM–7610 F, Japan) operated at 15 kV with a working distance of 15.5 mm. Before imaging, the samples were sputter-coated with carbon using a JEOL JEC-3000FC auto fine coater. Elemental composition was identified by energy dispersive X–ray spectroscopy (EDS) coupled to the FE-SEM. Further transmission electron microscope (TEM, Model: JEOL JEM–1400, USA) was employed to evaluate the internal structure, morphology, and crystal size of the sample due to its high resolution and high contrast imaging. For this, samples were dispersed in ethanol and sonicated for 15–20 min then after drop-cast onto a TEM grid, and air-dried. TEM images were recorded at an accelerating voltage of 120 kV. Ultraviolet-visible (UV–Vis) absorption spectrum was recorded using a fiber optic spectrometer (AvaSoft-3648, Avantes, Netherlands), equipped with a portable UV–Vis light source, sample chamber, and charge-coupled device (CCD) detector. A small amount (0.05 mg) of TiO_2_ NPs powder was dispersed in 4 ml of double–distilled water, and spectrum was acquired in the 200–800 nm range with a resolution of 2.3 nm and an integration time of 20 s. To identify various functional groups and bands in the synthesized TiO_2_ NPs, a Fourier transform infrared (FT–IR) spectrophotometer (Perkin Elmer Buckinghamshire, UK) was utilized in the range of 4000–500 cm^−1^ with a resolution of 4.0 cm^−1^. The sample was prepared as a KBr pellet. For the antioxidant activity assay, a UV–Vis spectrophotometer (Spectra Max, San Jose, CA, USA) was used to measure absorption at 517 nm for DPPH radical scavenging activity.

### Determination of antioxidant activity

#### *DPPH* (2, *2–diphenyl–1–picrylhydrazyl) free radical scavenging activity*

The antioxidant efficacy of biogenically synthesized TiO_2_ NPs was evaluated via the DPPH radical scavenging assay and compared against the reference antioxidant, ascorbic acid. The potency was quantified through the half-maximal effective concentration (pEC_50_) and antioxidant radical power (ARP), parameters indicative of their radical-neutralizing capacity.

#### FRAP (ferric reducing antioxidant power) free radical scavenging activity

For the ferric reducing power (FRAP) assay, 10 µl of varying sample concentrations (0–1000 µM) and the reference antioxidant, ascorbic acid (SRL, Cat. No. 23006; 0–50 µg/ml), were combined with 0.04 ml of 0.2 M sodium phosphate buffer (Rankem, Cat. No. S0240; pH 6.6) and 0.05 ml of 1% potassium ferricyanide [K₃Fe(CN)₆] (SRL, Cat. No. 15766). The reaction mixture was thoroughly vortexed and incubated at 50 °C for 20 min, with untreated wells serving as controls. Post-incubation, 0.5 ml of 10% trichloroacetic acid (SRL, Cat. No. 92390) was added, followed by 50 µl of deionized water and 50 µl of 0.1% ferric chloride (Fisher Scientific, Cat. No. 23585). The absorbance of the resulting solution was recorded at 700 nm against the blank using a microplate reader (iMark, Bio-Rad). The IC_50_ value was subsequently calculated using GraphPad Prism 6 software in accordance with Eq. (1).

1 $$\:\%\:inhibition=\left(\frac{{A}_{Sample}-{A}_{Control}}{{A}_{Control}}\right)\times\:100\:$$

Where *A*_*Sample*_ represents the absorbance of the test sample and *A*_*Control*_ denotes the absorbance of the control.

### Statistical data analysis

All antioxidant assays were conducted in triplicate, and the data are presented as mean ± standard deviation (SD). Statistical and graphical analyses were performed using MS Office Excel 2021, OriginPro 2024b, and ImageJ software.

## Results and discussions

### X–ray powder diffraction study

**Fig. 2:**
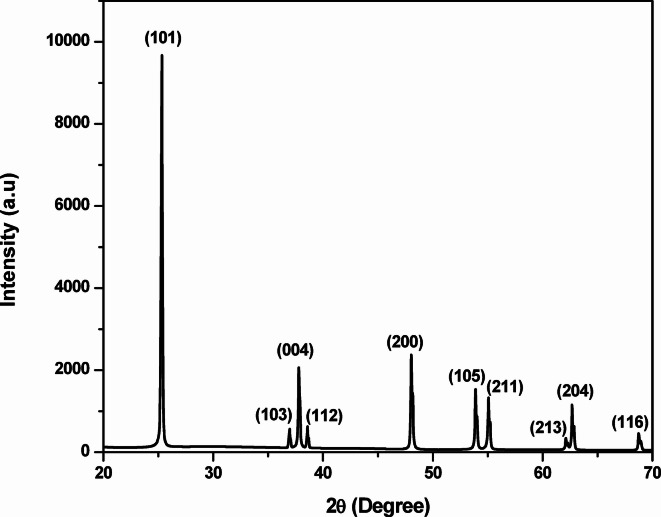
XRD pattern of the green-synthesized TiO_2_ NPs.

Figure 2 shows the XRD pattern of the synthesized TiO_2_ NPs. All diffraction peaks were indexed to the (101), (103), (004), (112), (200), (105), (211), (213), (204), and (116) planes corresponding to 2θ angles of 25.3°, 36.9°, 37.8°, 38.6°, 48.0°, 53.9°, 55.2°, 62.2°, 62.7°, and 67.9°, respectively. The obtained peaks indicate that the product was the crystalline structure of the pure anatase TiO_2_ phase (JCPDS File No. 86–1157). The anatase phase belongs to the tetragonal crystal system with a space group*I4*_*1*_*/amd*(141). The calculated lattice parameters were a = b = 3.783Å and c = 9.497Å^44^.These results confirm the formation of high-purity anatase phase TiO_2_ NPs.

X–ray diffraction is a standard method for estimating particle size for polycrystalline powder or a single crystal. The crystallite size of the green-synthesized TiO_2_ NPs was evaluated using the Scherrer Eq. (2)^45^.

2 $$\:D=\frac{K\lambda\:}{\beta\:\text{cos}\theta\:}$$

Where K is the Shape factor, λ is the X–ray wavelength (1.54 Å), β is the full width at half maximum (FWHM) in radians, and θ is the Bragg angle. The Scherrer constant denotes the shape of the particle, and its value is most commonly taken as 0.9. Using the most intense diffraction peak, the crystallite size was calculated using the above Eq. (2) as 57.9 nm, as also tabulated in Table 1.

**Table 1:** XRD data of the green-synthesized TiO_2_ NPs.

S.No.	2θ (degree)	Miller indices	FWHM(β) (degree)	FWHM(β) (radians)	βcosθ	4sinθ	Crystallite size (D)in nm
**1**	25.3	(101)	0.1469	0.002563889	0.002502	0.8756	57.9 (by Scherrer equation)
**2**	36.9	(103)	0.2058	0.003591888	0.003407	1.2660	
**3**	37.8	(004)	0.2073	0.003618068	0.003423	1.2956	
**4**	38.6	(112)	0.2098	0.003661701	0.003456	1.3220	
**5**	48.0	(200)	0.2184	0.003811799	0.003482	1.6268	
**6**	53.9	(105)	0.2368	0.004132940	0.003684	1.8128	67.6 (by W-Hplot)
**7**	55.2	(211)	0.2408	0.004202753	0.003724	1.8532	
**8**	62.2	(213)	0.2567	0.004480260	0.003836	2.0660	
**9**	62.7	(204)	0.2599	0.004536111	0.003874	2.0812	
**10**	67.9	(116)	0.2707	0.004724606	0.003919	2.2340	

### Williamson–hall (W–H) plot

The Scherrer equation estimates crystallite size from the XRD peak broadening but does not account for lattice imperfections such as defects, stacking faults, grain boundaries, and strain. To overcome these limitations, the W–H plot has been widely applied to estimate both crystallite size and lattice strain^46^. This approach considers the combined effect of micro-strain and crystal size on peak broadening. For calculating particle size, the strain value (ε) is given by the Cauchy Eq. (3).

3 $$\:\beta\:\text{cos}\theta\:=\frac{\lambda\:}{D}+4\epsilon\:\text{sin}\theta\:$$

Where β = full width at half maximum (FWHM) in radians; λ = X-ray wavelength; θ = Bragg angle; D = crystallite size; and $\epsilon$= lattice strain.

**Fig. 3:**
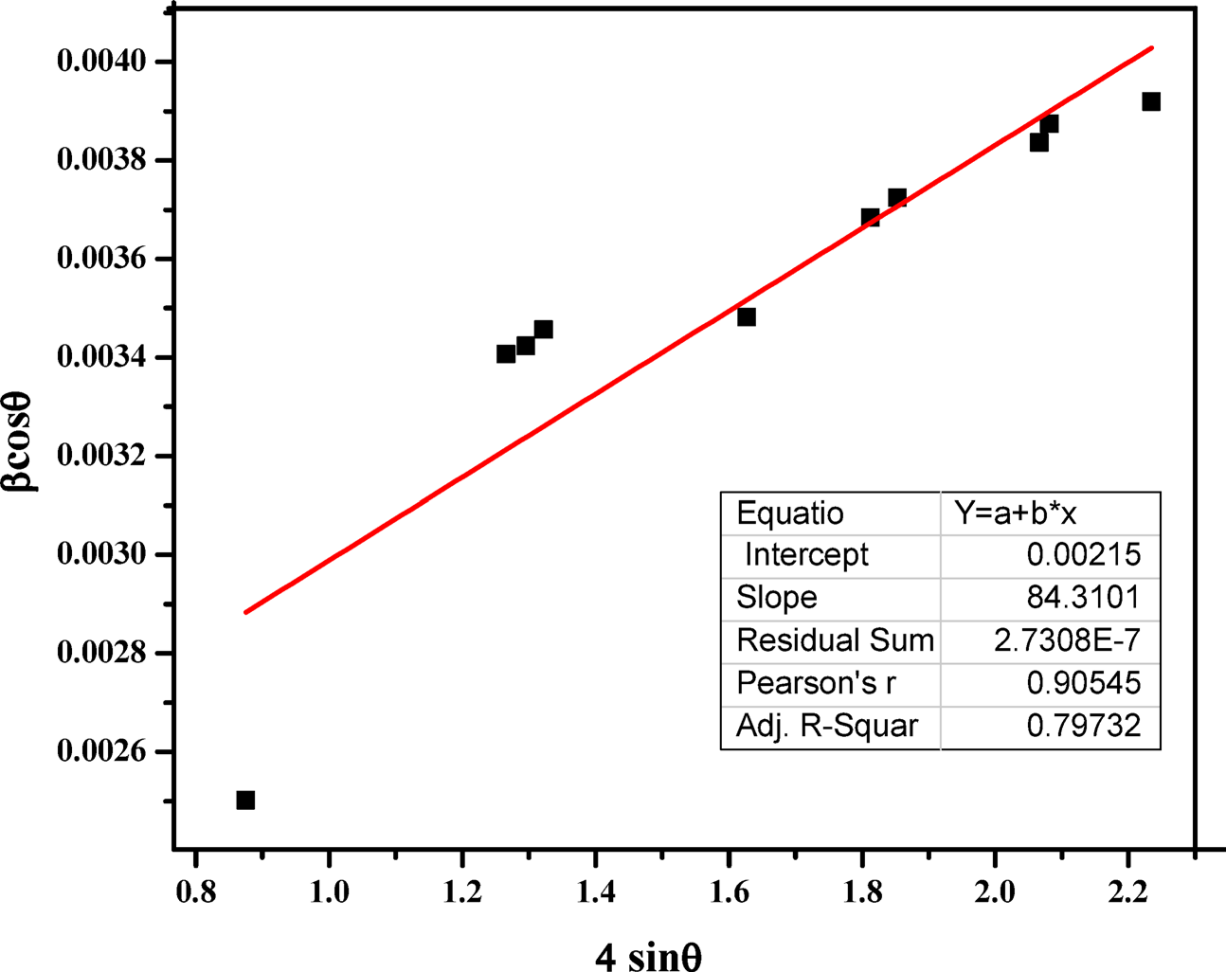
Williamson–Hall (W–H) plot for the green-synthesized TiO_2_ NPs.

Figure3 shows the W–H plot for the green-synthesized TiO_2_NPs, where the βcosθ was plotted against 4sinθ using XRD data from Table 1. The slope of the linear fit gives the strain (ε), and the y-intercept provides$\:\:\frac{\lambda\:}{D}\:$ from which the crystallite size is calculated. From the W-H analysis, the lattice strain and crystallite size are calculated. The analysis yielded a lattice spacing of 0.00084 and the average crystallite size of 67.6 nm. These values are consistent with the size obtained from the Scherrer equation, confirming the reliability of the W-H method^47^. In this study, the uniform deformation model (UDM) was applied because anisotropic strain effects are difficult to quantify in the presence of organic ligands. The UDM thus provides a more reliable estimate of crystallite and strain, consistent with the characteristics of TiO_2_ NPs synthesized via a green route, while the Scherrer method assumes zero strain. The W-H analysis accounts for strain, giving a more accurate result for highly strained materials^48^. If discrepancies occur, TEM can be used to validate W-H results, as TEM provides direct imaging of individual nanocrystallites. To ensure accuracy, instrumental broadening correction should be applied during XRD analysis to avoid over estimating peak broadening and to extract reliable size and strain values^49^.

### FE–SEM analysis

**Fig. 4:**
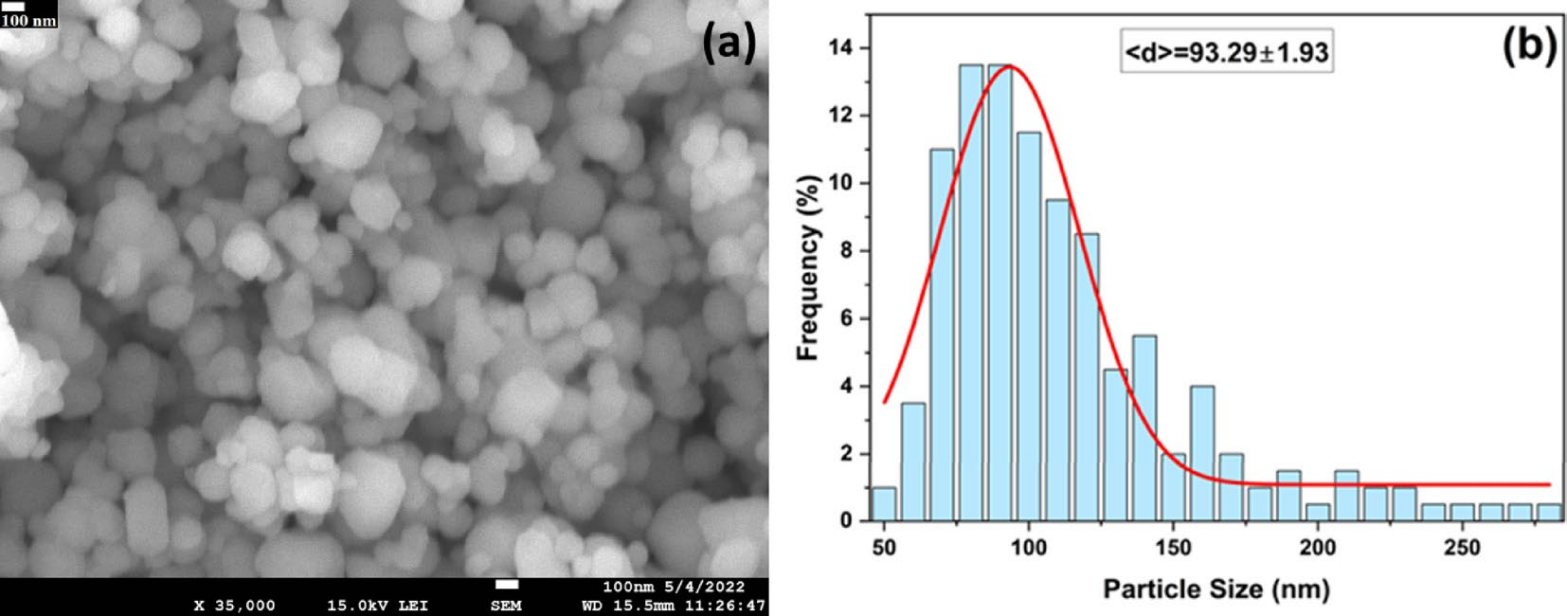
(**a**) FE–SEM micrograph, (**b**) Particle size histogram of the green-synthesized TiO_2_ NPs.

FE-SEM image and the particle size histogram of green-synthesized TiO_2_ NPs are shown in Figs. 4(a) and 4(b). The morphology of TiO_2_ NPs was found spherical with little agglomeration and uniformly distributed, which is comparable to an earlier report^42^. After examination of the FE–SEM micrograph as depicted in Fig. 3(a), the size (diameter) of spherical TiO_2_ nano-particles (taken as a total of 200 spherical shape particles) were measured using Image J software, and the data were plotted as a size distribution histogram (Fig. 3(b)). The average particle diameter (< d>) was 93.29 nm with a standard deviation of 1.93 nm. The mean particle size obtained from the FE-SEM/TEM was larger than the crystallite size estimated from XRD, which can be attributed to the aggregation or oriented attachment of multiple crystallites, forming larger nanostructures^50^.

### EDX spectrum analysis

**Fig. 5:**
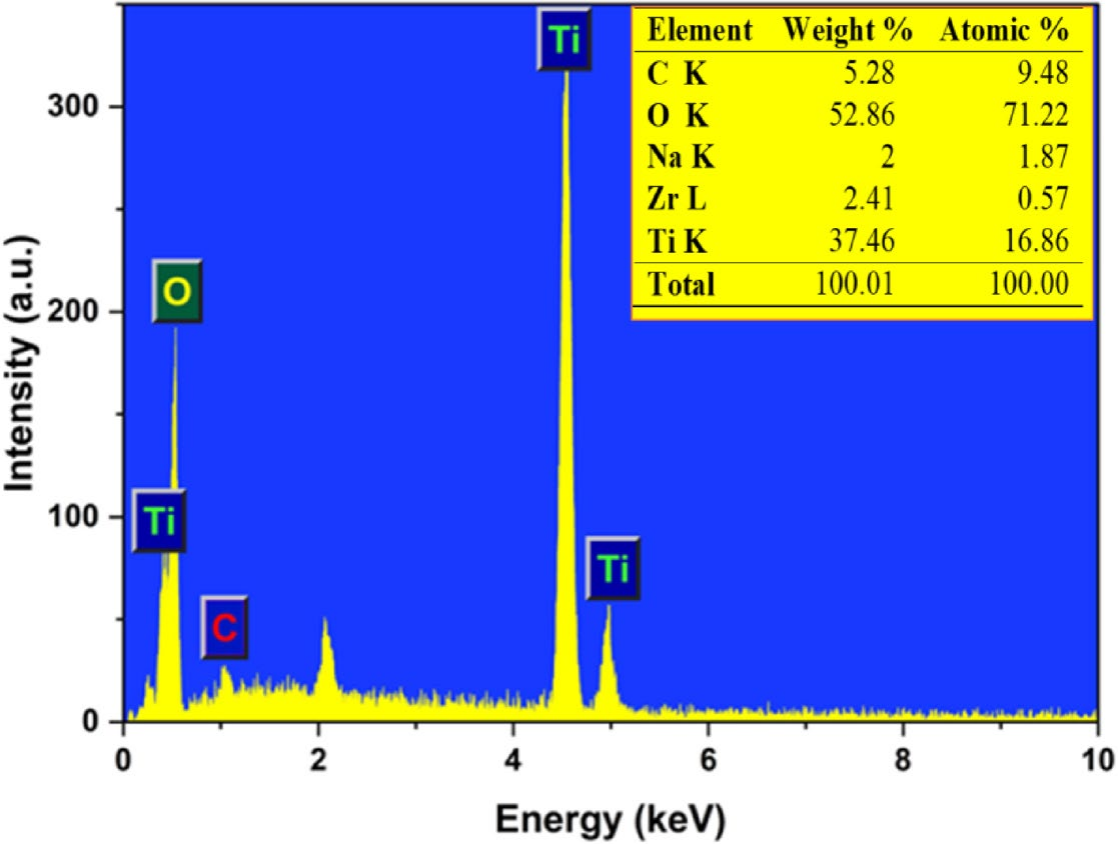
EDX spectrum of the green-synthesized TiO_2 _NPs.

The EDX spectrum of the green-synthesized TiO_2_ NPs represents the elemental composition of the materials. Figure 5 indicates the presence of elements titanium (Ti) and oxygen (O), and the inset table demonstrates a high atomic percentage of oxygen (71.22%) along with titanium (16.86%). Subsequently, confirming the formation of TiO₂ NPs. The presence of carbon (5.28 wt%), Sodium (2.00 wt%), and zirconium (2.41 wt%) in the inset table may be the experimental set or peel extract. The sharp and well-defined peaks of Ti at approximately 0.5 keV and 4.5 keV further confirm the successful formation of the titanium oxide matrix^42^.The carbon peak suggests the presence of surface-adsorbed organic compounds, which is consistent with capping or stabilizing agents from the extract. Trace elements such as Na and Zr may originate from the natural mineral content of the plant material or the extraction medium^51^. Their presence on the NPs surface can influence surface charge, photocatalytic behavior, and interaction with biological systems. Although such bio-derived residues may enhance dispersibility or bioactivity of the TiO_2_ NPs, their uncontrolled presence can compromise reproducibility and optical performance^52^. Therefore, the NPs were subjected to additional purification, such as ethanol washing, and mild calcinations at 100–200 °C to reduce surface-bound residues.

### TEM analysis

**Fig. 6:**
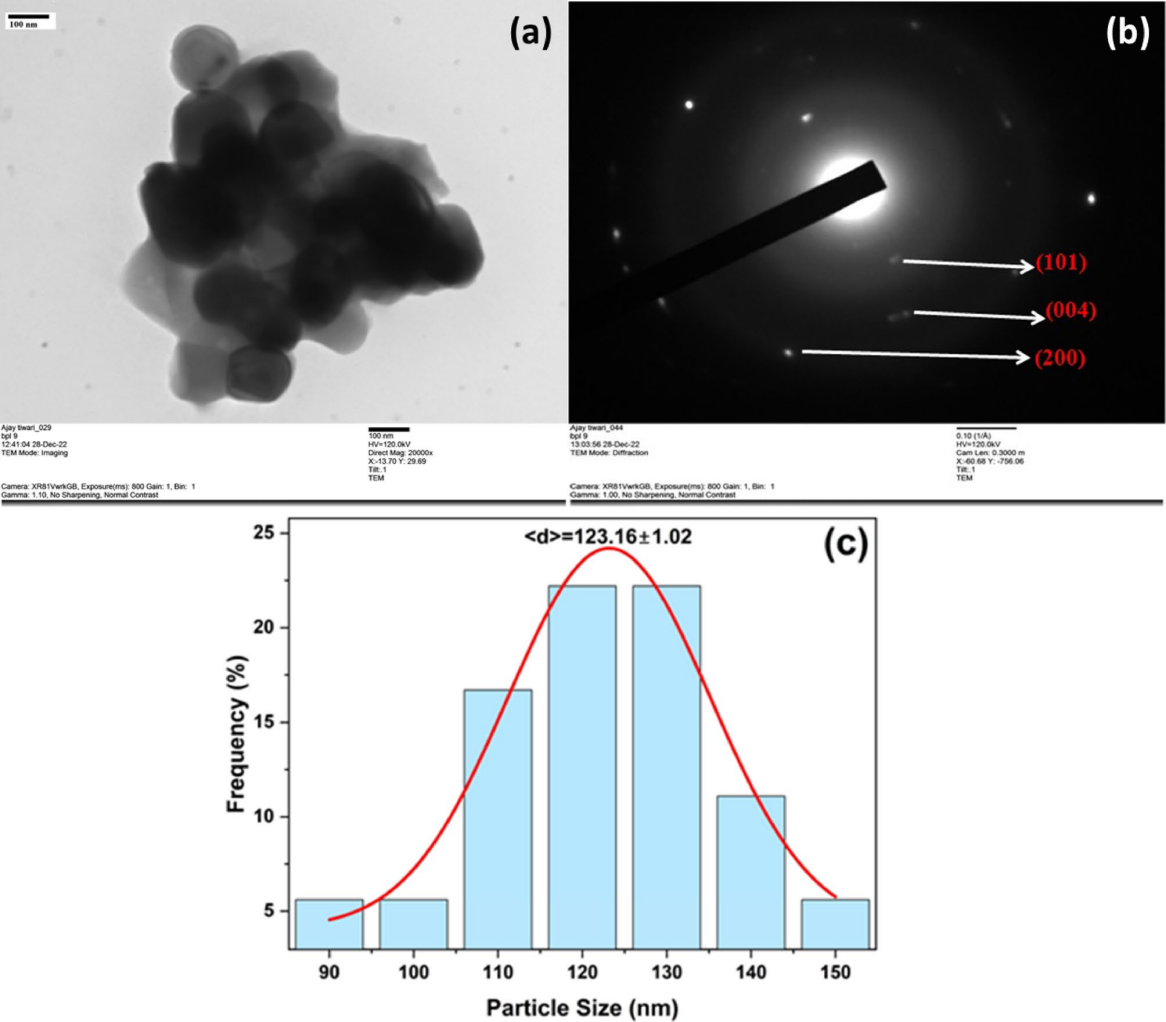
(**a**) TEM micrograph, (**b**) the corresponding SAED pattern containing the diffraction spots, and (**c**) Histogram of particle size distribution of the green-synthesized TiO_2_ NPs.

The TEM micrograph of green-synthesized TiO_2_ NPs is shown in Fig. 6(a). The selected area electron diffraction (SAED) pattern (Fig. 6b) shows diffraction spots, and the ring pattern confirms the nanoscale size and crystalline nature of the TiO_2_ NPs. The crystalline phase of the NPs may be accurately determined by measuring the distance of these spots from the center, which represents the crystallographic interplanar d-spacing. The SAED of Fig. 6(b) was employed for the analysis of d spacing, and a measurement showed that these spots’ distances ranged between 0.33, 0.25, and 0.19 nm. The (101), (004), and (200) planes of the anatase phase TiO_2_ depicted in Fig. 6(b) were found to be well matched by these d-spacings. The corresponding SAED pattern confirmed that the green synthesized TiO_2_ NPs were crystalline (Fig. 6(b)). The obtained SAED pattern was well consistent with the XRD images. It is visible in the TEM image that almost all the TiO_2_ NPs are spherical with little agglomeration^42^. After the examination of TEM micrographs, the average size of TiO_2_NPs (shown in Fig. 6(c)) was measured by line profiling on the diameter of each nanoparticle in the TEM images. Then, a nanoparticle size histogram (~ 85 NPs were analysed) was made. The histogram with a non-linear Gaussian fit shown in Fig. 6(c) reveals that the average size (< d>) of the TiO_2_ NPs is 123.12 nm with a standard deviation of 1.02 nm. The increase in grain size observed in TEM results may be due to the ageing of the TiO_2_ NPs over a long time.

### UV–Visible study

**Fig. 7:**
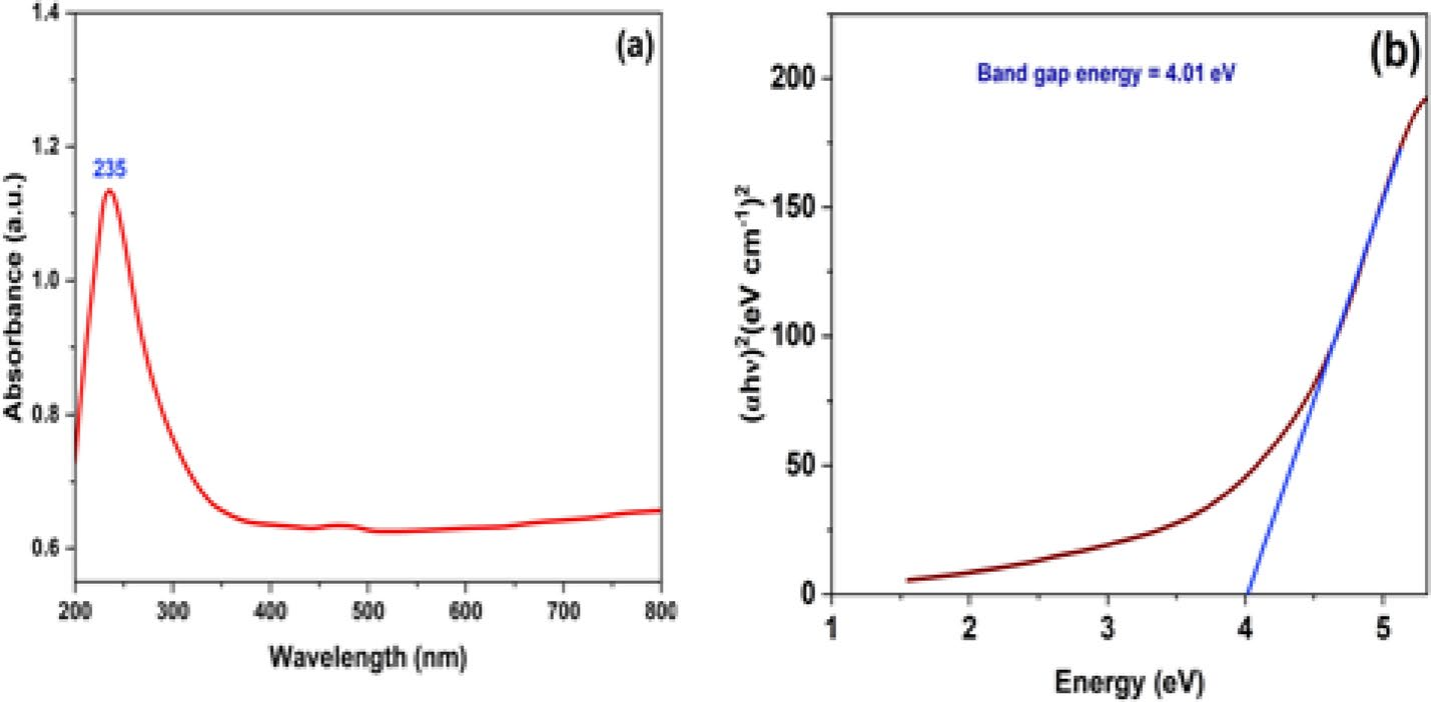
(**a**) UV–Vis absorption spectrum of the green-synthesized TiO_2 _NPs and (**b**) Tauc plot to determine the band gap energy of green-synthesized TiO_2 _NPs.

The UV–Vis absorbance spectrum and corresponding Tauc plot of green synthesized TiO_2_ NPs are shown in Fig. 7(a) and 7(b), respectively. The absorbance peak at 235 nm confirmed the formation of TiO_2_ NPs. The band gap energy was calculated from the Tauc plot^53^ by extrapolating the linear portion of (αℎυ)^2^ versus photon energy (ℎυ), was 4.01 eV, slightly higher than the reported value^54^. Rehman et al. (2025) investigated the optical properties of green-synthesized TiO_2_ NPs using *citrus* lemon juice, and observed an absorbance peak at 315 nm^35^. Similarly, Narh et al. (2024) synthesized Ag-TiO_2_ NPs using *citrus sinensis* peel extract and found a strong absorbance band at 442 nm^55^. Sakeel and his colleague (2025) also confirmed the formation of TiO_2_ NPs at room temperature through titanium ion reduction with a visible colour change as an indicator. Generally, green synthesized TiO_2_ NPs from plant sources exhibit absorbance bands in the wavelength range of 245–265 nm^56^.

The presence of residual organic compounds in green-synthesized TiO₂ NPs, typically derived from phytochemicals such as flavonoids, polyphenols, and terpenoids present in plant extracts, can significantly influence their optical characteristics. These biomolecules often remain adsorbed on the nanoparticle surface, introducing additional absorption features in the UV-Vis spectrum and the band gap^57^. While the intrinsic band gap of TiO_2 _NPs is typically around 3.2 eV, which is slightly less than the above-estimated band gap (4.01 eV). It may be due to organic capping agent interference^58^.

### FT–IR study

**Fig. 8:**
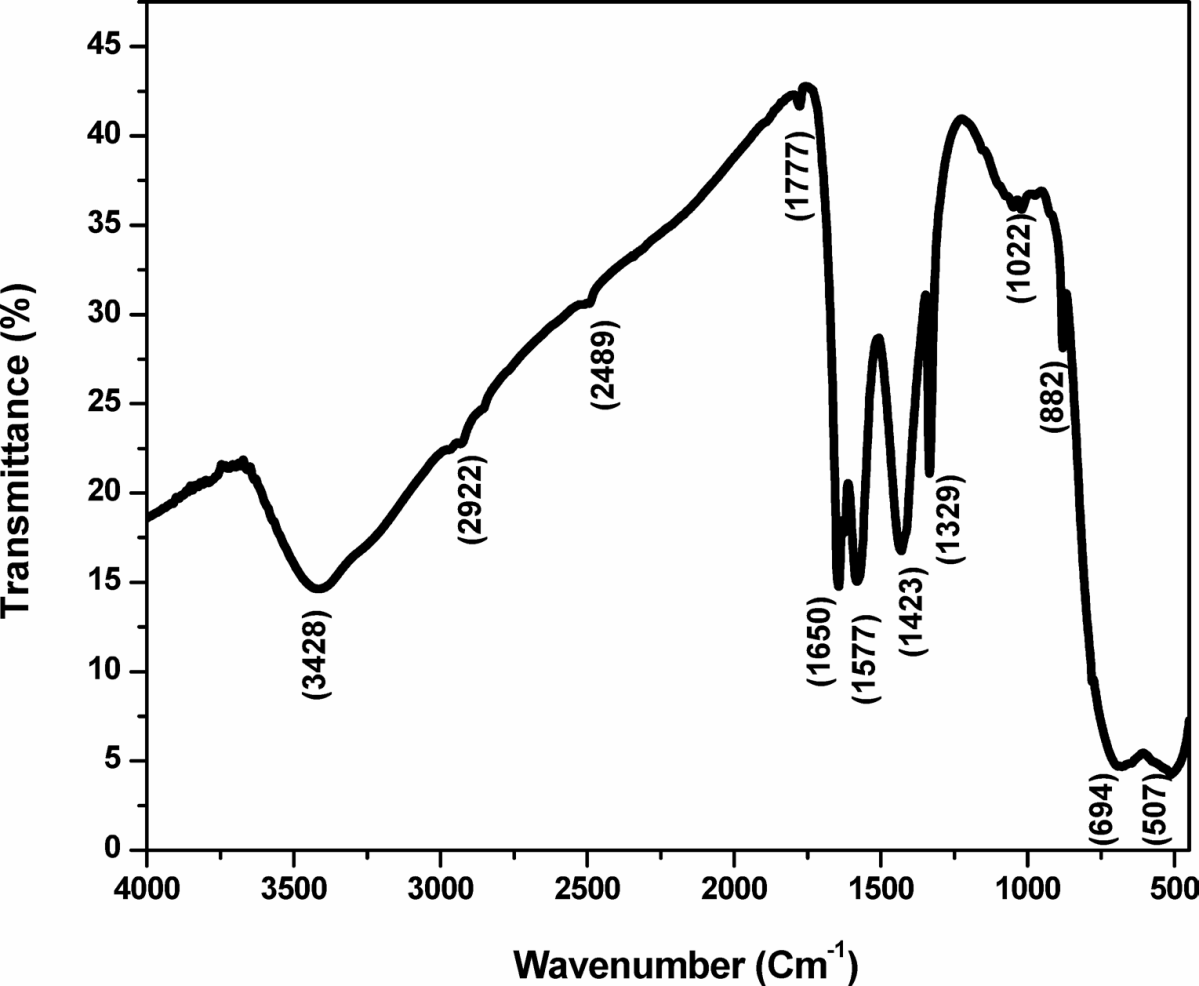
FT–IR spectrum of the green-synthesized TiO_2_ NPs.

The FT–IR spectrum of green-synthesized TiO_2_ NPs was recorded within the wavelength range of 4000–500 cm^−1^ to identify the functional groups, responsible for the production and stabilization of the NPs (Table 2; Fig. 8). Several characteristic peaks confirmed the presence of functional groups derived from the Kinnow peel extract. The strong, broad and intense band at 3428 cm^−1^ reflects the O-H stretching vibration of the hydroxyl group representing moisture. The peak having medium intensity at 2922 cm⁻¹ showed C–H stretching, whereas 2489 cm⁻¹ indicates the H-bonded O–H stretching of carboxylic acids. The strong absorption at 1777 cm⁻¹ assigned to C = O stretching of carbonyl groups, confirmed the presence of aldehydes, ketones, or esters in the peel extract. The peaks at 1650 and 1577 cm^−1^ correspond to C = C stretching of alkenes groups, indicating potential contributions from proteins and aromatic phyto constituents. The band at 1423 cm⁻¹ is due to O–H bending (phenolic groups), while the peak at 1329 cm⁻¹ corresponds to -C–N bending vibration band of amine groups. The absorption peak obtained at 1022 cm⁻¹ is due to C–O stretching vibrations of polyphenols and flavonoids, which play a key role in the capping and stabilization of the NPs. The peaks observed between 882 and 507 cm⁻¹ correspond to Ti–O and Ti–O–Ti stretching vibrations, confirming the successful formation of TiO₂ NPs^59^. Previous studies have also shown approximately similar results. Rahman et al. (2025) reported the broad O-H, N-H, Ti-OH, and Ti-O-Ti stretching vibration at 3237, 2163, 1623, and 630 cm^-1^, respectively, for TiO_2_ NPs synthesized using *citrus* lemon juice extract^35^. Similarly, Amanulla et al. (2019) observed peaks at 3220, 2930, 2349, 1391, 1050, and 700 cm^-1^corresponding to vibration of water molecules, N-H and C = O group, fatty acid, carbohydrate, protein, and Ti-O-Ti/Ti-O stretching vibration^60^.

**Table 2:** Band assignment/Functional group in FTIR spectrum of the green synthesized TiO_2_ NPs.

S.*N*.	Wave number (cm^−1^)	Band assignment	Functional group
1	3428	O-H stretching vibration	Hydroxyl (-OH)
2	2922	C-H stretching vibration	Aliphatic hydrocarbons
3	2489	O-H stretching (H-bonded)	Carboxylic acids
4	1777	C = O stretching	Carbonyl
5	1650	C = C stretching/Amide I band	Alkenes/Aromatic ring
6	1577	C = C stretching/Amide II band	Aromatics/Amides
7	1423	O-H bending vibration band	Phenolic
8	1329	-C-N bending vibration band	Amine
9	1022	C–O stretching vibration	Polyphenols/flavonoids
10	882	C–H out-of-plane bending	Aromatic/Metal oxygen
11	694	Ti-O-Ti stretching	Metal Oxygen (Ti-O bond)
12	507	Ti-O-Ti/Ti-O stretching vibration	Metal Oxygen vibration

## Antioxidant activities of TiO_2_ NPs

### Determination of antioxidant activity using the DPPH free radical scavenging activity (RSA) method

The antioxidant activity of green-synthesized TiO₂ NPs was assessed through the DPPH radical scavenging assay. A primary stock suspension was prepared by dispersing 50 mg of TiO₂ NPs in double-distilled water, and subsequent serial dilutions were performed to achieve concentrations ranging from 1 to 8 mg/m in 96-well microtiter plates.

The ascorbic acid, dissolved in deionized, double-distilled water, was used as a standard drug. DPPH is a stable free radical that facilely dissolves in methanol, producing a deep violet solution with a characteristic absorbance at 517 nm^61,62^. In this assay, the DPPH solution prepared in methanol was mixed separately with the TiO_2_ NPs suspension and the ascorbic acid solution. Thereafter, the mixture was incubated at room temperature for 30 min. A gradual colour change from deep violet to translucent or pale yellow, the scavenging of DPPH radicals by antioxidants. Higher concentrations of NPs exhibited a faster and more pronounced conversion. The reaction endpoint was determined when no further colour change occurred.

A blank was prepared under identical conditions without test samples, while methanol was used as a reference. The absorbance was recorded at 517 nm using a SpectraMax UV–Vis spectrophotometer. The free radical scavenging activity (RSA) in % of both drug samples was evaluated using the formula (1)^63,64^. All the experiments were performed in triplicate. The calculated free RSA (%) value for different concentrations (1–8 mg/ml) of both the drug samples, namely ascorbic acid and TiO_2_NPs, are tabulated in Table 3.

**Table 3:** DPPH free RSA (%) of ascorbic acid and TiO_2_ NPs with different concentrations.

Concentration of drug (mg/ml)	Free RSA (%) against DPPH	
	Ascorbic acid	TiO_2_ NPs
**1**	16.550 ± 0.01	21.265 ± 0.51
**2**	27.574 ± 0.56	23.959 ± 0.66
**3**	39.470 ± 0.55	51.099 ± 0.70
**4**	49.828 ± 0.04	48.672 ± 0.052
**5**	62.608 ± 0.30	49.951 ± 0.21
**6**	71.680 ± 0.33	72.706 ± 0.16
**7**	92.378 ± 0.51	79.058 ± 0.99
**8**	98.229 ± 0.22	94.384 ± 0.78

Note: values are in Mean ± Standard Deviation of three independent experiments.

#### Half maximal response concentration or efficiency concentration (pEC_50_)

**Fig. 9:**
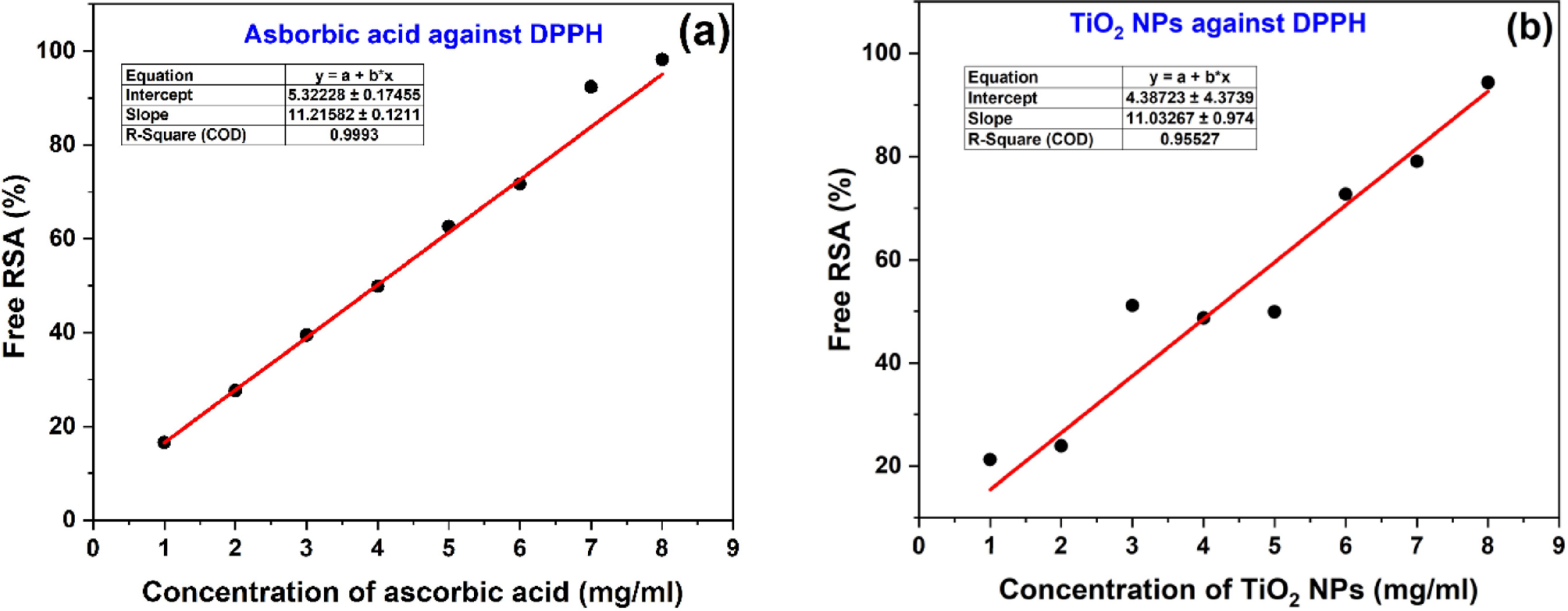
Plot of concentration of drug vs. DPPH Free RSA (%) of (**a**) Ascorbic acid, and (**b**) TiO_2_ NPs.

Half maximal response concentrations of ascorbic acid and TiO_2_ NPs were calculated from the graph of “% free RSA” plotted against the “drug sample concentrations.” Fig. 9(a) represents the plot of different concentrations of ascorbic acid vs. DPPH Free RSA (%), while Fig. 9(b) shows the plot of different concentrations of TiO_2_ NPs vs. DPPH Free RSA. The linear fit of the above scatter plot data gives the value of slope (b) and intercept (a) on the y–axis, and the pEC_50_was calculated by^65^:

$$\:y=a+b\times\:xorx=(y-a)/b$$

$$\:\Rightarrow\:EC_{50}=\:(50-c)/b$$

**Fig. 10:**
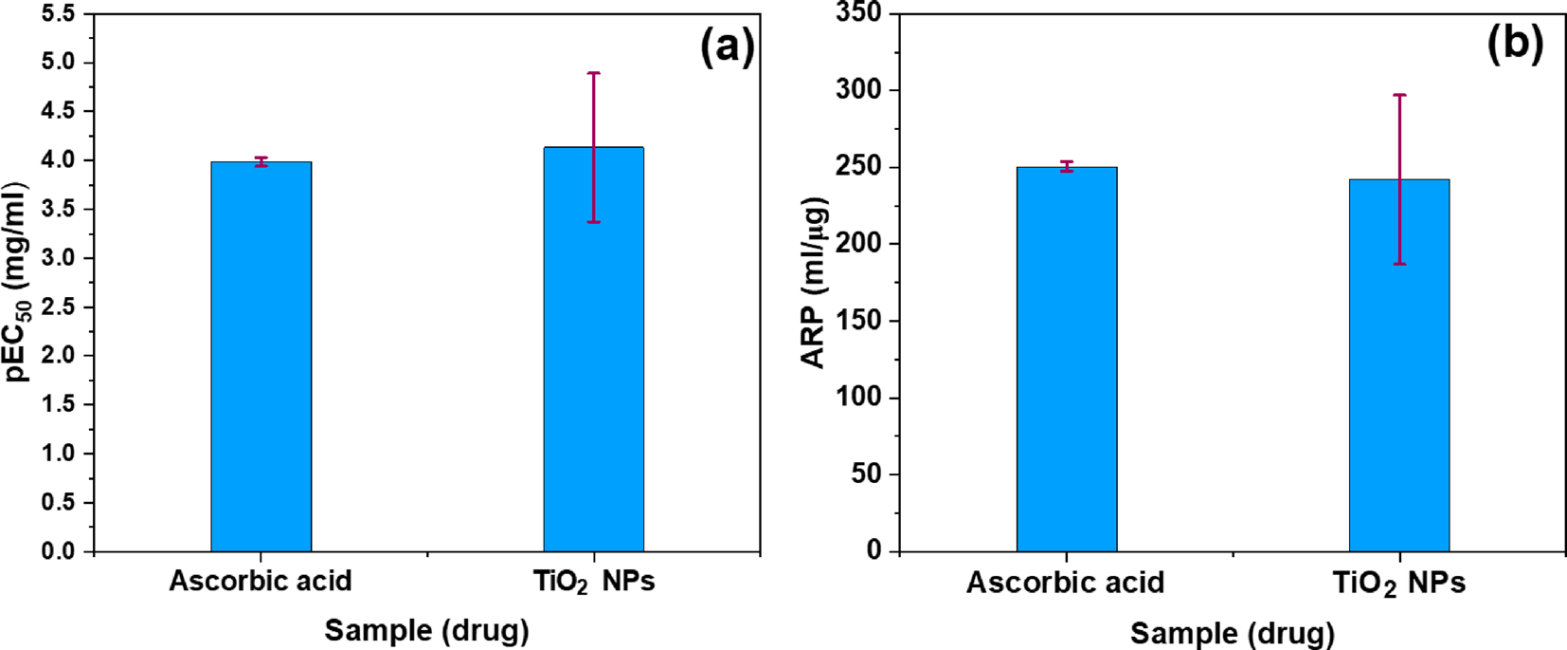
Bar graph of (**a**) pEC_50_ value and (**b**) ARP value of ascorbic acid, and TiO_2_ NPs.

The pEC_50_ serves as a measure of compound potency and is expressed in mg/ml, or µg/ml which is used as the unit of measure. The calculated pEC_50_ values were found to be 3.986 ± 0.046 mg/ml for the ascorbic acid drug and 4.131 ± 0.761 mg/ml for the TiO_2 _NPs drug, and are also tabulated in Table 4. A bar graph of the pEC_50_ value of both drug samples is shown in Fig. 10 (a).

**Table 4:** pEC_50_ and ARP values of ascorbic acid and TiO_2_ NPs.

Sample (drug)	pEC_50_ (mg/ml)	ARP (ml/µg)
Ascorbic acid	3.986 ± 0.046	250.60 ± 2.90
TiO_2_NPs	4.131 ± 0.761	242.07 ± 55.00

#### Antioxidant radical power (ARP)

The potency of a drug can be measured by the reciprocal of its pEC_50_ value. The reciprocal of pEC_50_ may be called antioxidant radical power^66^, and the most potent drug is the one with the lowest pEC_50_ value or greatest ARP value. Thus, the ARP of TiO_2_ NPs and ascorbic acid was evaluated by the formula (4):

4 $$\:\text{A}\text{R}\text{P}=\frac{1}{{\text{p}\text{E}\text{C}}_{50}}\times\:100$$

The ARP values were found to be 250.60 ± 2.90 ml/µg and 242.07 ± 55.00 ml/µg for the ascorbic acid drug and TiO_2_ NPs drug, respectively. These values are also presented in Table 4 and their comparison is illustrated in Fig. 10 (b). It is evident from the Fig. 10b; Table 4, the ARP value (242.07 ± 55.00 ml/µg) of the TiO_2_ NPs drug is nearly equal to the ARP value (250.60 ± 2.90 ml/µg) of the standard ascorbic acid drug. This finding, demonstrates that TiO_2_ NPs exhibit comparable potency to ascorbic acid. Based on their pronounced antioxidant activity, TiO_2_ NPs were selected for the development of a formulation incorporating herbal antioxidants and free radical scavengers^67^.

In this study, the DPPH radical scavenging activity of Kinnow peel extract–mediated TiO₂ nanoparticles across a broad concentration range demonstrated remarkable antioxidant efficacy, comparable to that of ascorbic acid, a standard reference compound. Free radicals, generated in the human body through exposure to radiation, toxic chemicals, and tobacco smoke, possess unpaired electrons, making them highly reactive and capable of accelerating oxidative reactions. Oxygen is the most important free radical in the human body. When oxygen-free radicals are exposed to radiation, electrons are removed from other molecules, damaging DNA and other cellular components. Due to this, some persistent diseases are caused, like cancer, heart problems, muscle failure, and diabetes^68^. Antioxidants act against free radicals and destroy them by acting as powerful remedies and saving cells from damage^69,70^. In laboratories, it is proven that antioxidants help in the prevention of cancer^71^. Generally, NPs of metallic salt show an excellent antioxidant effect^72^, among which the antioxidant effects of TiO_2_ NPs have been reported well. Although various *citrus* fruit peels have been explored for the synthesis of TiO_2_ NPs but the Kinnow peel aqueous extract based synthesis of TiO_2_ NPs has not been previously reported to the best of my knowledge.

### Evaluation of antioxidant potential by FRAP assay

The antioxidant efficacy of green-synthesized TiO₂ NPs was assessed through the ferric reducing antioxidant power (FRAP) assay, a widely employed method to estimate the electron-donating capacity of compounds in mitigating oxidative stress-induced damage^73^. The findings revealed that TiO₂ NPs prepared via the green synthesis approach demonstrated notable antioxidant potential.

**Fig. 11:**
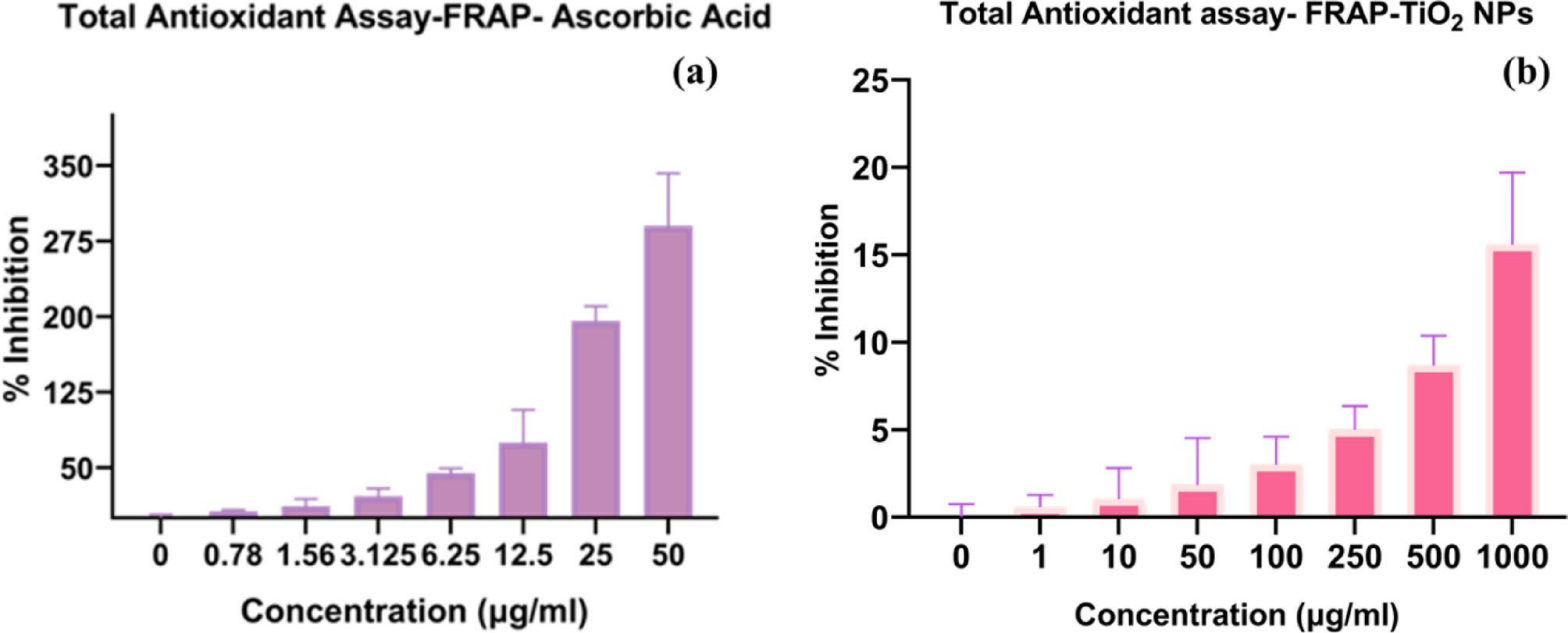
% inhibition of free radicals (**a**) ascorbic acid (standard), (**b**) green synthesized TiO_2_ NPs.

The results confirmed that green-synthesized TiO_2_ NPs exhibit strong antioxidant potential. Figure 11(a and b) shows the % inhibition activity of ascorbic acid (standard) and green-synthesized TiO_2_ NPs. The antioxidant performance of the TiO_2_ NPs improves with increasing concentration (1, 10, 50, 100, 250, 500, and 1000µg/ml). At higher concentrations, the nanoparticle demonstrated greater scavenging activity against free radicals. This trend aligns with findings from a previous study, showing the reliability of the results^74^. Overall, Green synthesized TiO_2_ NPs exhibited superior antioxidant capacity in both assays.

## Conclusions

A facile, cost-effective, and eco-friendly method for the synthesis of TiO₂ NPs using Kinnow peel extract was successfully developed. The bioactive compounds present in the kinnow peel extract act as a natural reducing and stabilizing agent for the synthesis of TiO_2_ NPs. A comprehensive characterization was carried out, which showed crystallite size of 57.9 nm, spherical morphology, tetragonal structure, a distinct absorbance at 235 nm, and high stability. The calculated optical band gap of 4.01 eV indicates suitability for various optical applications. FT-IR spectroscopy confirmed the presence of several phytochemical moieties over TiO_2_ NPs.

Moreover, the antioxidant potential assessed through DPPH and FRAP assays confirmed the pronounced free radical scavenging ability of the green-synthesized TiO₂ NPs. To establish their safety and therapeutic relevance, additional investigations focusing on in vitro cytotoxicity and in vivo biocompatibility are required. Beyond biomedical applications, exploring their photocatalytic, antimicrobial, and environmental remediation properties could further expand the scope of green-synthesized TiO₂ NPs, enhancing their translational significance in both healthcare and environmental sustainability.

## Data Availability

Most of the data generated or analysed in this study are included in this article. Additional data sets supporting the findings are available from the corresponding author, Dr. Abhimanyu Kumar Singh, and the first author, Dr. Ajay Kumar Tiwari, upon reasonable request.
